# 

**Published:** 1841-05

**Authors:** 


### WESTERN JOURNAL

OF

### MEDICINE AND SURGERY.

### COLLABORATORS.

Edward H. Barton, M. D. *Professor of the Theory and Practice of Medicine, in the Medical College of Louisiana.*

William J. Barbee, M. D. *Cincinnati, Ohio.*

George W. Bayless, M. D. *Demonstrator of Anatomy in the Louisville Medical Institute.*

A- H. Buchanan, M. D. *Columbia, Tennessee.*

Charles Caldwell, M. D. *Professor of the Institutes of Medicine and Medical Jurisprudence in the Louisville Medical Institute.*

Samuel A. Cartwright, M. D. *Mississippi.*

A. Clapp, M. D. *New Albany, Indiana.*

Jedediah Cobb, M. D. *Professor of Anatomy in the Louisville Medical Institute.*

John Esten Cooke, M. D. *Professor of the Theory and Practice of Medicine in the Louisville Medical Institute.*

Samuel D. Gross, M. D. *Professor of Surgery in the Louisville Medical Institute.*

John Hardin, M. D. *Munfordsville, Ky.*

John P. Harrison, M. D. *late Professor of Materia Medica, in the Cincinnati College.*

John F. Henry, M. D. *Bloomington Illinois*

Samuel P. Hildreth, M. D. *Marietta, Ohio.*

Samuel Hogg, M. D. *Nashville, Tennessee.*

M. Z. Kreider, M. D., *Lancaster, Ohio.*

Moses L. Linton, M. D. *Springfield, Kentucky.*

Henry Miller, M. D. *Professor of Obstetrics and the Diseases of Women and Children in the Louisville Medical Institute.*

John W. Monette, M. D. *Washington, Mississsippi.*

Samuel B. Richardson, M. D., *Lecturer on Anatomy and Surgery, &c, Louisville, Ky.*

John L. Riddell, M. D. *Professor of Chemistry in the Medical College of Louisiana.*

Landon C. Rives, M. D. *late Professor of Obstetrics in the Medical Department of the Cincinnati College.*

Charles W. Short, M. D. *Professor of Materia Medica in the Louisville Medical Institute.*

G. Troost, M. D. *Professor of Chemistry and Mineralogy in the University of Nashville.*

Amasa L. Trowbridge, M. D. *Professor of Surgery, Willoughby University, Ohio.*

John A. Warder, M. D. *Cincinnati, Ohio.*

William Wood, M. D. *Cincinnati, Ohio*

BIBLIOGRAPHICAL NOTICE.

The Dublin Dissector, or Manual of Anatomy, &c. &c., together with the Elements of Pathology. By Robert Harrison, A. M. M. B. T. C. D. &c.; with additions by Robert Watts, Jr. M. D., Professor of Anatomy in the College of Physicians and Surgeons in the city of New York. 12mo: pp. 541. New York, Philadelphia, and Boston: 1840.

The above forms the title page to a work which we have recently met with, and which, we believe, is the fourth edition of the "Dublin Dissector" that has issued from the American press.

In so far as the text is purely a descriptive anatomy, we have always considered it one of the best dissecting room manuals which we possess—that the arrangement is very well adapted to the convenience of the student, presenting as it does, a description of the different parts in the order in which they are met with on dissection—and that the style is perspicuous, and perhaps not too prolix for the patient laborer. But candor compels us to add, that we consider those parts of the work referred to as "Elements of Pathology," as superfluous and burdensome. At the table, the student wants a description of the parts which he is dissecting, as concise as is compatible with perspicuity; and any attempt to draw the mind from what should be the special and exclusive objects of his study at the time, by giving an account (however short) of the different accidents and morbid changes to which they may be liable, serves only to produce confusion, by multiplying the objects of attention. If he be systematic in his labors, he wants to study *Healthy Anatomy* alone in the dissecting room; and give Surgery and Morbid Anatomy his whole attention at other times.

The additions which the American editor has made, consist in a classification of the muscles, according to the parts on which they act; which, we think, will greatly tend to "impress upon the minds of students their number, situation, and functions"—in an account of the weights and measures of the different organs; which is perhaps the most valuable "addition," unless it be the "varieties" of the arterial system—in the "varieties" of the muscular, arterial, and venous systems —a list of the muscles arising from, and inserted into, each individual bone, in connection with its description—an enumeration of the various fractures to which the bones, and dislocations to which the articulations, are liable—and an extension of the "Elements of Pathology." Against these latter may of course be urged the same objection that was mentioned in reference to the text. The parts of the book, however, which we deem objectionable, do not so materially detract from its value, but that we consider it, all in all, one among the best works that we have upon the subject.

G. W. B.

### RECEIPTS FOR THE MEDICAL JOURNAL,

*For the month ending April* 30, 1841.

| | |
|---|---|
| Dr. D. E. Gibson, Belmont, Tennessee, | $10 00 |
| J. H. Gibson, Berlin, Ill., | 5 00 |
| Prof. Dickson, Charleston, S. C., | 10 00 |
| Dr. J. B. Wall, Wallonia, Ky., | 10 00 |
| T. P. Tayler, Gosport, Ia., | 10 00 |
| W. P. Golliday, Dixon's Mills, Ia., | 2 00 |
| Drs. J. Sutcliffe & Co., Bloomfield, Ky., | 5 00 |
| Dr. J. D. Alexander, Marrow Bone, Ky., | 5 00 |
| J. Carter, Bloomingdale, Ohio, | 5 00 |
| T. B. Johnson, Glasgow, Ky., | 2 50 |
| W. D. Jourdan, do do, | 2 50 |
| A. E. Geohegan, Elizabethtown, Ky., | 5 00 |
| B. C. Wood, Carrollton, Ill., | 5 00 |
| English, Jacksonville, Ill., | 5 00 |
| Helm, Springfield, Ill., | 10 00 |
| Drs. King & Barnes, Decatur, Ill., | 10 00 |
| Dr. W. Kellar, Shelbyville, Ill., | 5 00 |
| Q. C. Alexander, Vandalia, Ill., | 5 00 |
| T. Booth, Clarksville, Mo., | 5 00 |
| Hogden, Campbellsville, Ky., | 5 00 |
| Shuttlesworth, do do, | 5 00 |
| Hiestan. do do, | 5 00 |
| J. Morrison, Arcadia, Ill., | 5 00 |
| G. A. Conn, Leiper's Fork, Tenn., | 5 00 |
| W. M. Dodson, Waynesville, Mo., | 5 00 |
| John Baty, Vincennes, Ia., | 5 00 |
| Osborn, Rural Hill, Tenn., | 5 00 |
| J. Motley, Hamburgh, Tenn., | 5 00 |
| B. W. Avent, Murfreesboro, Tenn., | 10 00 |
| W. Long, New Maysville, Ia., | 5 00 |
| A. C. McLaughlin, Tremont, O., | 5 00 |
| J. McCan, West Milton, O., | 5 00 |
| J. Wells, Buffaloe Grove, Ill., | 5 00 |
| G. B. Thompson, Rome, Ia., | 5 00 |
| S. Goslee, Sr., Hawesville, Ky., | 10 00 |

### TO DELINQUENTS.

You will see, gentlemen, that the paying list has very much fallen off and that there is already room on the same page to commence the publication of your names; but feeling satisfied that you will rush in to fill up the page with payments, we still forbear to commence the black list. Upon looking over our subscription book, we find there are a great number who have not even paid for the first year. This is too bad; and we shall hesitate a little before we make public the names of any of these. We know that we have fulfilled all we ever promised, and having great faith in human nature and particularly in our subscribers, we believe that they have delayed remitting because they thought from the length of the paying list that it was not necessary in order to make it respectable, and perhaps they were apprehensive that we could not find room for them all at once. There *was* something in this, but they must now see the necessity of coming to the rescue.

☞ Remit by mail, at our risk, under the frank of the Postmaster; who, under the regulations of the P. O. Department, will in all cases frank letters containing subscription money.

☞ No subscription to commence but with the commencement of a volume. No subscription can be discontinued until the end of the year, and none, after one number of a given volume has been mailed, until the end of the volume.

PRENTICE & WEISSINGER.

---

The Western Journal of Medicine and Surgery is published monthly by the undersigned, at the corner of Main and Fifth streets, Louisville, at $5 per annum, payable in advance. Each number contains from 80 to 84 pages making two volumes in the year of about 500 pages.

Contributions to its pages by the Physicians of the Valley of the Mississippi, addressed to the Editors, are respectfully solicited.

Letters on business, to be addressed, postage paid, to the publishers.

☞ Postmasters, by the regulations of the Postoffice Department, will frank letters containing subscription money, and all remittances so franked are at the risk of the publishers.

*July* 25, 1840. PRENTICE & WEISSINGER

